# How to engage patient partners in health service research: a scoping review protocol

**DOI:** 10.1186/s40900-021-00268-z

**Published:** 2021-04-26

**Authors:** Sarah Cecilie Tscherning, Hilary Louise Bekker, Tina Wang Vedelø, Jeanette Finderup, Lotte Ørneborg Rodkjær

**Affiliations:** 1 Research Centre for Patient Involvement (ResCenPI), Aarhus University, Aarhus, Central Denmark Region Denmark; 2 Leeds Unit for Complex Intervention Development (LUCID), Leeds Institute of Health Sciences, School of Medicine, University of Leeds, Leeds, UK; 3 Department of Neurosurgery, Aarhus University Hospital, Aarhus, Denmark; 4 Department of Renal Medicine, Aarhus University Hospital, Aarhus, Denmark; 5 Department of Infectious Diseases, Aarhus University Hospital, Aarhus, Denmark

**Keywords:** Patient engagement, Patient and public involvement, Patient participation, Patient partner, Impact, Health service research, Scoping review protocol

## Abstract

**Background:**

The patients’ and the carers’ roles in health service research has changed from being solely participants in studies to also being active partners and co-designers in the research process. Research carried out with or by patient partners is an increasingly accepted component of health service research in many countries, but how researchers can best approach engaging patient partners in the research process is still not clear. There is a need for guidance to support researchers when engaging patient partners and assess how such engagement impacts on research outputs. The aim of this paper is to present a protocol for a scoping review of published literature on how to engage patient partners effectively in the research process. Investigating this aim implies examining: a) how to engage patient partners in the research process; and b) what impact such engagement has on research outputs. This scoping review protocol is the first to examine how to engage patient partners effectively across different diseases and research areas.

**Methods:**

A scoping review using a systematic process informed by Arksey and O’Malley’s framework will be carried out across six electronic databases using the terms ‘patient participation’, ‘community participation’, ‘research personnel’, ‘patient and public involvement’ and ‘patient partner’. We will include published reviews concerning engagement of patient partners in the research process in healthcare settings, and exclude studies assessing engagement in treatment and healthcare. Two reviewers will screen the titles and abstracts of articles independently for inclusion, and extract data from articles that meet the inclusion criteria. Where there is disagreement, a third reviewer will be consulted to facilitate consensus. The data elicited will include: author and study characteristics; research aims and findings; description of patient engagement in the research process; and assessment impact. Descriptive data and narrative analysis will synthesize findings.

**Discussion:**

To understand how to engage patient partners effectively in the research process, the impact of such engagement must be taken into consideration to give a qualified suggestion for future guidance. We hope this review will raise awareness of which common elements constitute effective engagement of patient partners in the research process.

## Plain English summary

Engaging patients and carers in health service research is an increasingly common approach to promoting and exploring patients’ perspectives on all stages of the research process. In this protocol we argue that there is an issue concerning how researchers can best engage patient partners. The aim of this protocol is to examine the most effective way to engage patient partners across different research areas and patient groups.

### Methods

A scoping review method brings together other people’s research findings. We will identify relevant literature through a systematic search of electronic databases and correspondence with colleagues and experts. We will include published studies in healthcare settings and concerning the engagement of patient partners in the research process, and exclude studies focusing on patient engagement in treatment and healthcare services. Two reviewers will screen the titles and abstracts of articles independently for inclusion; a third reviewer will resolve discrepancies. We will extract data to categorise the different types of engagement within the studies included and what difference engaging patient partners makes. The findings can provide guidance to researchers, patient partners, carers and decision makers when planning to engage patient partners in their projects.

### Discussion

To understand how to engage patient partners in the research process, we must consider what difference engaging them makes to a research project. We hope this review will raise awareness of which common elements constitute effective engagement of patient partners in the research process across different patient groups and different research areas.

## Background

The patient’s role in health service research has changed from being solely a participant in studies to also being an active partner and co-designer in the research process. Greenhalgh et al. [1] highlight three important aspects of this shift. Firstly, patients have the right to be engaged in contributing to the research agenda, and researchers have a moral duty to ensure their engagement. Secondly, patient perspectives improve the value and quality of research by integrating their lived experience as recipients of healthcare into research design and delivery. Thirdly, engaging patient partners in the research team increases the accountability, relevance and transparency of the research [1]. Today, many international initiatives have patient engagement on their agenda, but with different understandings of how to engage patient partners [2]. Four different approaches from government-funded organizations are compared here: the INVOLVE initiative was founded in 1996 in the United Kingdom and is part of the Centre for Evidence and Dissemination in the government agency the National Institute for Health Research (NIHR) [3]; the Canadian Institutes of Health Research (CIHR) established the Strategy for Patient-Oriented Research (SPOR) in 2001 [4]; the International Collaboration for Participatory Health Research (ICPHR) started in 2009 involving several countries [5]; the Patient-Centered Outcomes Research Institute (PCORI) was founded in the United States of America in 2012 [6]. Table 1 illustrates similarities and differences in their terminology. For example, where NIHR uses the term ‘public involvement’, CIHR uses ‘patient-oriented research’, ICPHR uses ‘participatory health service’, and PCORI uses ‘patient engagement’ [5, 7–9]. CIHR, ICPHR and PCORI emphasize engaging patient partners in the entire research process [5, 8, 9], whereas NIHR acknowledges different levels of involvement [10]. These initiatives set the scene for engaging patient partners in research today, but from our perspective, more clarity about their theoretical and empirical frameworks is needed.

**Table 1:** Four approaches to engaging patient partners in the research process

Organization	Term for patient	Approach	Definition of the approach
**NIHR, UK**	Public	Public involvement	“(…) research being carried out ‘with’ or ‘by’ members of the public rather than ‘to’, ‘about’ or ‘for’ them.” [7]
**CIHR, Canada**	Patient	Patient-oriented research	“(…) a continuum of research that engages patients as partners, focusses on patient-identified priorities and improves patient outcomes.” [8]
**ICPHR, international**	People	Participatory health research	“(…) the goal is to maximize the participation of those whose life or work is the subject of the research in all stages of the research process. (…) Research is not done ‘on’ people as passive subjects providing ‘data’, but ‘with’ them to provide relevant information for improving their lives.” [5]
**PCORI, USA**	Patients, patient partners	Patient engagement	“The meaningful involvement of patients, caregivers, clinicians, and other healthcare stakeholders throughout the entire research process – from planning the study to conducting the study, and disseminating study results.” [9]

Studies carried out with patient partners are increasingly accepted as a component of health delivery research in many countries, but it is still not clear how researchers can best engage patient partners in the research process and capture the impact of their engagement [11–14]. Mockford et al. [11] state that there needs to be a common understanding of what is meant by engaging patient partners in the research process and how this can be conceptualized. Domecq et al. [12] add that it is unclear who to engage or when, and how to perform this task. Brett et al. [13] state that examining the impact of patient partner engagement in the research process is essential to understand how such engagement works, for whom, why and in what circumstances. However, Staley [14] argues that knowledge about impact is often contextual and may not enhance our wider understanding of when, why and how engaging patient partners makes a difference. Our search in the JBI Database of Systematic Reviews and Implementation Reports showed that this is the first scoping review to examine how to engage patient partners effectively across different diseases by taking the question of impact into consideration. This paper addresses the evidence gaps on how to engage patient partners and assess the impact of their engagement across different health contexts. We hope this review can provide guidance for researchers, patient partners and decision makers when choosing how to engage patient partners for the benefit of the research process and research outcomes.

### Aim

The aim of this paper is to present a protocol for a scoping review of published literature on how to engage patient partners effectively in the research process. Investigating this aim implies examining: a) how to engage patient partners in the research process; and b) what impact such engagement has on research outputs.

### Context and team composition

This work is being carried out in the context of the Research Centre for Patient Involvement (ResCenPI) [15]. ResCenPI has 70+ members carrying out research into the design, implementation and evaluation of complex interventions to improve people’s engagement in healthcare relevant to their daily lives across all health contexts [15]. One core research area is to examine methods for engaging patient partners effectively in the research process and explore the consequences for the patient, carer, health professional, researcher and study. SCT has been employed as a patient partner member and a research assistant at ResCenPI to identify the focus of this review, TWV and JF are researchers affiliated to ResCenPI, LØR is deputy lead, and HLB lead of ResCenPI. Given SCT is a patient partner and has limited research experience, the author group’s composition aims to bring together diverse researchers with: a) expertise in scoping review methods and engaging patient partners (JF, LØR, HLB); and b) practical experience as health professionals at Aarhus University Hospital (TWV, JF, LØR). While all authors of this protocol are patients or carers to some extent, two authors are active contributors to research and health services delivery in their roles as a patient (SCT) and as an informal carer (JF). The roles of the two patient partners (SCT, JF) have actively impacted on the team’s idea generation, formulation of the research area and method, and reflection on key issues from their perspectives as patient partners.

## Methods

The study design is guided by Arksey and O′Malley’s [16] Scoping Review Framework, and Preferred Reporting Items for Systematic reviews and Meta-Analysis extension for Scoping Reviews (PRISMA-ScR) is used as a framework [17]. The review method will be a scoping review because we need to identify key concepts of effective engagement to provide evidence to inform practice [18]. We want to: a) identify and map the available evidence concerning effective engagement of patient partners; and b) identify research gaps in exploring patient partners’ contributions to the research process [18]. This protocol documents the process of planning the work and will aid reflection at each stage.

### Stage 1: identification of the research question

We are undertaking the scoping review to answer the following research question:

*How can patient partners be engaged effectively in the research process?*

To answer this research question, we will examine: a) how to engage patient partners in the research process; and b) how their engagement impacts on research outputs. The key concepts within the research question are presented in Table 2 and include the terms ‘patient partners’, ‘engagement’, ‘effective’ and ‘research process’, inspired by PCORI [9], CIHR [8], Staley [14], and Sweeney and Morgan [19].

**Table 2:** Defining the key concepts within our research question

Concept	Definition
Patient partners	‘Patient’ refers to a person, or a carer to such a person, with a lived experience such as an injury, illness or disease [9]. ‘Partner’ indicates the active and equal contribution of patient expertise and other research team stakeholders. A ‘patient partner’ is a patient who collaborates actively with other stakeholders in as many stages and areas of the research process as possible and relevant.
Engagement	Meaningful and active collaboration among stakeholders where patient partners are engaged in any research process [8].
Effective	The most relevant, transparent and successful engagement, assessed by how patient partners are engaged in the research process. The impact can be captured via different aspects of the research process, such as research agenda, research design and delivery, research ethics, people engaged, researchers, participants, wider community, community organizations, and implementation and change [14].
Research process	All possible stages of the research, from planning and delivery to interpreting and disseminating the results [19].

There are many discussions relevant to defining and understanding different concepts in this area, e.g. Harrington et al. [20]. We will not go further into these discussions, but instead focus on the aim of our study, which is to examine how to engage patient partners most effectively in the research process.

### Stage 2: identification of relevant studies

#### Search methods

The electronic databases Medline (PubMed), CINAHL, PsycInfo, Scopus and Embase (including Cochrane reviews), and Google Scholar will be searched. The search strategy will utilize search terms (keywords/subject headings) that relate to our key concepts (Table 2) with the Boolean term ‘OR’ to combine the search terms within a concept. We have chosen to connect the terms only with ‘OR’ given our broad research question. The first author has collaborated with a search specialist Helene Sognstrup to develop the search strategy and will engage with her when planning the final search stages. Table 3 presents a sample search strategy. We will include articles found through expert advice and hand searching the key articles. We will additionally search for grey literature in the JBI Database of Systematic Reviews and Implementation Reports.

**Table 3:** Sample search strategy for Medline

Search terms connected with ‘OR’	
(‘Patient participation’).mp	
(‘Community participation’).mp	
(‘Research personnel’).mp	
Patient and public involvement	
Patient partner	

#### Inclusion/exclusion criteria

To answer our research question, we will include published reviews of empirical papers in healthcare settings concerning the engagement of patient partners in the research process. Studies assessing engagement in treatment and healthcare, and not in the research process, will be excluded.

### Stage 3: study selection

**Fig. 1:**
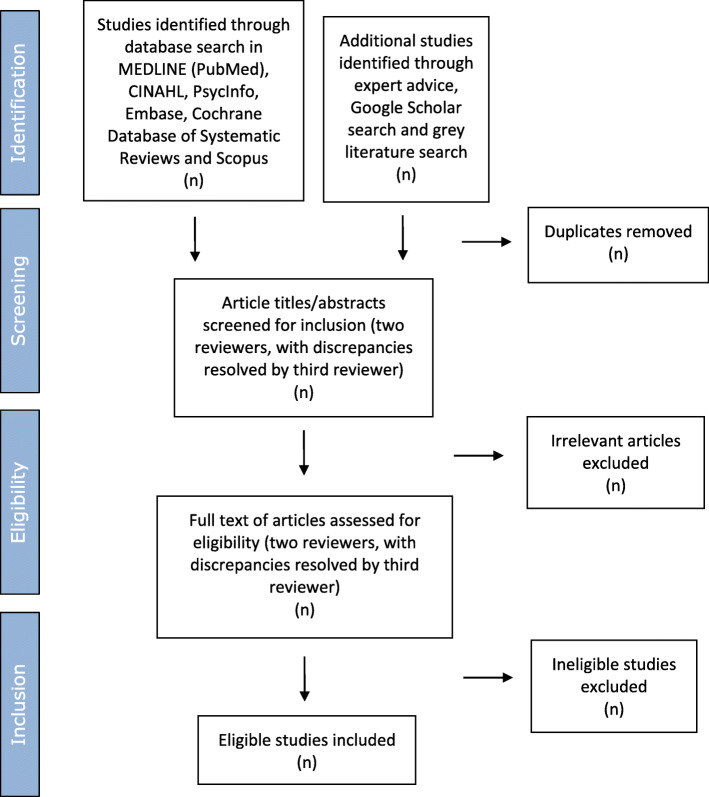
Flow of studies

The study selection process is illustrated in Fig. 1 [21]. The process consists of two steps:

1. Two reviewers will screen titles/abstracts in all eligible literature against the inclusion criteria. SCT will act as principal reviewer throughout, and TWV, JF and LØR will alternate as second reviewer. HLB will resolve discrepancies. We will record whether each article reviewed has been included or excluded and the primary reason for exclusion.
2. The full text of articles will be reviewed prior to data extraction to check their eligibility for inclusion. SCT will again act as principal reviewer, with TWV, JF and LØR alternating as second reviewer. HLB will be consulted on any disagreements. Primary reasons for exclusion will be recorded.

All authors will use the online application Covidence (Veritas Health Innovation, Melbourne Australia) to manage the selection of studies [22]. We will handle data using a combination of Excel and NVivo 12 [23].

### Stage 4: charting and extracting the data

The research team has identified a preliminary set of variables for inclusion in the data extraction form (Table 4). These variables are informed by our appraisal of the literature on the engagement of patient partners in research and the measurement of impact that has been used to develop this protocol. A data extraction form will be developed to elicit the same type of data from each article chosen [17]. All reviewers will extract data from the full text of articles included in the review, ensuring there are two data extraction forms for each paper.

**Table 4:** Variables for inclusion in the data extraction form

Theme	Variables
Characteristics of the authors and the study	Authors’ surnames Study location Study title Publication year
Characteristics of the research aim and findings	Aim of the study Research method Important results
Characteristics of the engagement	Context of engaging patient partners How/when patient partners have been engaged in the research process
Characteristics of the impact	Definition of impact Measured/resulted impact

### Stage 5: collating, summarizing and reporting the results

The extracted data will be collated and analysed using descriptive methods [17]. Our objective is to determine the most effective way to engage patient partners in the research process across different research areas and patient groups. We acknowledge that it may be difficult to create a generic model that will include all types of patient partners most effectively in all research areas, and we do not necessarily think ‘one size fits all’. However, to avoid assumptions, we think it is appropriate to examine the question of what effective engagement is from a broad perspective. We envision that these findings will have implications for the general research area of engaging patient partners, as well as practical implications for researchers, patient partners and decision makers, when determining how to engage patient partners effectively and which research projects to support. The implications might include: a) enhancing the benefits of engaging patient partners in research projects; and b) identifying components to measure the impact of interventions that engage patient partners.

### Stage 6: consultation

According to Arksey and O’Malley [16], this stage is optional, but we think it is highly relevant to us as authors to see our work from the perspectives of different stakeholders, to qualify the findings and the feasibility of the study. We will recruit a patient partner expert group on the basis of the recommendations by Leask et al. [24]. This expert group will consist of about eight different patients with experience as patient partners who will be consulted to help define the tasks and discuss the results, to ensure that, from a patient’s perspective, the outcomes are relevant [24]. The type of engagement will be reported using the GRIPP2 checklist [25]. A group of Danish researchers with different levels of experience of engaging patient partners will be consulted to discuss the literature included and synthesize findings.

## Discussion

Patient partners are currently engaged in research processes in many ways. But how do we know whether their engagement makes a difference, and what kind of difference does it make? The impact of engaging patient partners in the research process is the subject of a great deal of discussion and can be measured from numerous perspectives, e.g. the research agenda, the people involved, the researchers or the community [14]. To understand how to engage patient partners effectively in the research process, the impact of their engagement must be taking into consideration. ‘How to engage’ should be connected with ‘with what impact’ to give a qualified suggestion for future guidance. Staley [14] argues that the existing literature does not pay enough attention to the specific context around the engagement. As a consequence, it tends not to generate knowledge that is useful beyond the original context. When conducting the literature search, implications to consider could include whether a study is transparent or not about how patient partners were engaged or what impact the engagement had. Also, patients are not a homogeneous group, and evidence indicates that some groups are more able to inform the research process than others [2]. Our research will critically examine which common elements researchers must take into consideration to ensure that all patient partners have equal involvement in the research process [2]. On the basis of this discussion, we consider it crucial to generate nuanced and evidence-based knowledge about how to engage patient partners most effectively.

## Data Availability

Not applicable.

## Abbreviations

ICPHR: International Collaboration for Participatory Health Research
NIHR: National Institute for Health Research
PCORI: Patient-Centered Outcomes Research Institute
ResCenPI: Research Centre for Patient Involvement
SPOR: Strategy for Patient-Oriented Research
